# Helpful ways providers can communicate about persistent medically unexplained physical symptoms

**DOI:** 10.1186/s12875-018-0881-8

**Published:** 2019-01-16

**Authors:** Nicole Anastasides, Carmelen Chiusano, Christina Gonzalez, Fiona Graff, David R. Litke, Erica McDonald, Jennifer Presnall-Shvorin, Nicole Sullivan, Karen S. Quigley, Wilfred R. Pigeon, Drew A. Helmer, Susan L. Santos, Lisa M. McAndrew

**Affiliations:** 1 War Related Illness and Injury Study Center, Veterans Affairs New Jersey Health Care System, 385 Tremont Avenue, East Orange, NJ 07018 USA; 2 Edith Nourse Rogers Memorial VA Hospital, Bedford, 01730 MA USA; 3 Department of Psychology, Northeastern University, Boston, 02115 MA USA; 4 Center of Excellence for Suicide Prevention, Canandaigua VA Medical Center, Canandaigua, NY USA; 5 Department of Educational and Counseling Psychology, University at Albany, 1400 Washington Ave Ext, Albany, NY 12222 USA; 6 Department of Rehabilitation Medicine, New York University School of Medicine, New York, NY USA

**Keywords:** Patient provider communication, Patient provider relationship, Medically unexplained symptoms, Gulf war illness, Veteran, Qualitative

## Abstract

**Background:**

Communication between patients and providers about persistent “medically unexplained” physical symptoms (MUS) is characterized by discordance. While the difficulties are well documented, few studies have examined effective communication. We sought to determine what veterans with Gulf War Illness (GWI) perceive as the most helpful communication from their providers. Veterans with GWI, a type of MUS, have historically had complex relationships with medical providers. Determining effective communication for patients with particularly complex relationships may help identify the most critical communication elements for all patients with MUS.

**Methods:**

Two hundred and-ten veterans with GWI were asked, in a written questionnaire, what was the most useful thing a medical provider had told them about their GWI. Responses were coded into three categories with 10 codes.

**Results:**

The most prevalent helpful communication reported by patients was when the provider offered acknowledgement and validation (*N* = 70). Specific recommendations for managing GWI or its symptoms (*N* = 48) were also commonly reported to be helpful. In contrast, about a third of the responses indicated that nothing about the communication was helpful (*N* = 63). There were not differences in severity of symptoms, disability or healthcare utilization between patients who found acknowledgement and validation, specific recommendations or nothing helpful.

**Conclusions:**

Previous research has documented the discord between patients and providers regarding MUS. This study suggests that most patients are able to identify something helpful a provider has said, particularly acknowledgement and validation and specific treatment recommendations. The findings also highlight missed communication opportunities with a third of patients not finding anything helpful.

## Background

Providers consider medically unexplained symptoms and syndromes (MUS) or persistent physical symptoms (PPS) to be among the most difficult conditions to treat [1–4]. MUS or PSS (also termed functional somatic syndromes, for discussion on terminology see [5–9]) are umbrella terms that describe chronic, often disabling, physical symptoms and syndromes. Conditions that fall under this umbrella include: fibromyalgia, chronic fatigue syndrome and *Gulf War Illness*. MUS are difficult to treat because there is not consensus among patients, providers or the medical community about the nature and best treatments for MUS [10, 11].

There is consensus that an essential component of treatment is effective communication between patients and health care providers [12] that allows them to negotiate concordant beliefs about MUS and develop shared treatment plans [10]. Patients recognize the importance of effective communication with their provider, describing improving communication as the most important goal, surpassing functional improvement [13].

Unfortunately, patient-provider communication about MUS is characterized by discordance. It has been described as a “tug of war” with each party trying to “pull” the interaction towards their own view rather than working together to develop a shared understanding of treatment and prognosis [14]. Providers report feeling frustrated and describe their interactions with patients with these symptoms and syndromes as “heartsink” [15] and “difficult” [16]. Patients describe feeling invalidated and discounted by their providers, for example being told their problems are “in their head” [17].

While the difficulties in patient-provider communication for MUS are well documented, what patients perceive as effective communication is not fully understood. In a qualitative study, Peters and colleagues found patients with MUS wanted explanations, support and the opportunity to share their problems [18]. When asked about the most helpful explanations, Salmon and colleagues found that patients sought explanations that were tangible and actionable, not judgmental [19]. These studies suggest that not all interactions between patients and providers are negative and describe what patients *wish* for in communication. It would be useful to also learn what patients have *found* to be helpful in their communication with providers. What patients *wish* for and what they *find* to be helpful are not always the same. Such knowledge would give providers concrete and specific statements they could use with their patients and would improve our understanding of effective communication for patients with MUS generally.

Identifying effective communication is particularly important for veterans with Gulf War Illness (GWI) who have documented distrust of the medical community and providers. GWI, a type of MUS, is characterized by chronic, disabling physical and neurocognitive symptoms and was the signature illness after Operation Desert Shield/Storm [20]. Upon return from combat, veterans with GWI described being marginalized by the medical community who they generally felt viewed GWI as a psychological condition and who under-appreciated the severity of GWI [21–24]. Some veterans were also concerned that the government was covering up the cause GWI, further complicating communication with medical providers [24]. Understanding the factors that contribute to effective communication among those with particularly complex relationships with their medical providers may suggest the most important components of communication for all patients with MUS.

In the current study, we asked veterans with GWI to describe the most useful information a medical provider has told them about their GWI. We also examined if there were differences in the severity of symptoms, disability and healthcare utilization between patients who found some communication helpful as compared to those who did not identify anything helpful. We hypothesized that those who did not identify anything helpful would have more severe symptoms, disability and greater healthcare utilization (i.e., more severe GWI).

## Methods

This analysis is from the baseline assessment from a randomized controlled trial comparing Problem-Solving Treatment (PST) to Health Education (NCT02161133; 06/11/2014). The study is on-going and the primary results have not yet been published. Institutional Review Board (IRB) approvals and other research oversight approvals were obtained. All participants gave informed consent. The study was funded by the Veterans Administration Clinical Sciences Research and Development (VA CSR&D).

### Patient characteristics

Subjects included the first 210 Gulf War Veterans (GWVs) with GWI recruited into the clinical trial [25]. Inclusion criteria included: deployment to Operation Desert Storm/Shield, Gulf War Illness (GWI) according to the Kansas definition [26], and scores at least a half a standard deviation worse than the population mean on the World Health Organization Disability Schedule II (WHO-DAS 2.0) [27]. To meet the Kansas definition of GWI, subjects had to present with symptoms in at least three (3) of the six (6) domains that consisted of fatigue, pain, neurological/cognitive mood, skin, gastrointestinal, and respiratory.

Exclusion criteria included: current suicidal/homicide intent or plan as determined using the Columbia Suicide Severity Rating Scale; schizophrenia or current psychotic symptoms; self-reported diagnosis of a degenerative brain disorder or serious psychiatric or medical illness (e.g. cancer in past year) which may limit generalizability of the findings, limit safety, or account for symptoms of GWI; and a disability that would preclude telephone use.

### Materials

The current analysis is from the baseline assessment. Within a written survey on healthcare utilization, patients were asked “what has been the most useful thing a medical provider has said regarding your GWI.” There was no limit to the patient’s response. All responses from this item were coded to identify common themes.

Patients also completed:

1. World Health Organization Disability Schedule (WHO-DAS 2.0) [28, 29]. The WHO-DAS 2.0 measures disability which is due to physical and mental health conditions with higher scores indicating less disability.
2. The patient health questionnaire somatic subscale (PHQ-15) which captures severity of physical symptoms. Scores above 15 are considered high, 10 to 14 medium, 5 to 9 low, and 0 to 4 no/mild [30].
3. Healthcare utilization was adapted from the National Health Interview Survey [31, 32]. We asked about frequency of seeing a primary care practitioner in the past year. Items were scored on a Likert scale from 0 to 5 (0 = no visits, 1 = 1 visit, 2 = 2–3 visits, 3 = 4–9 visits, 4 = 10–12 visits, 5 = 13+ visits).

### Coding procedures

The coding of responses was completed in two phases. In the first phase, three coders (FG, JP-S & NS) reviewed the responses and created 12 codes into which responses were placed. In the second phase, two new coders (DL & NA) reviewed the 12 codes and subject responses. They condensed the 12 codes into 10 codes which were organized into three larger categories; (1) acknowledgement and validation: (2) specific recommendations for managing GWI or its symptoms (3) nothing a provider has said has been helpful.

Following the establishment of this coding schema, two coders (DL & NA) individually coded 40 additional responses to establish interrater reliability. In this initial coding, approximately 80% of the coding was consistent across both raters and consensus was achieved in 100% of responses after a brief discussion.

Once reliability was obtained, the same two coders each individually coded the remaining responses (total *n* = 181) based on the previously determined coding schema; 87% of coding was consistent across both raters and 100% was consistent after a brief discussion. Some responses were not valid: 42 responses were removed from final coding due to either being intentionally/unintentionally skipped, endorsed as “not applicable”, or reported “don’t have GWI”. Statements could have more than one code if the response included multiple distinct codes.

### Quantitative analysis

After completing the coding, ANOVA analyses were conducted to determine if those who said that nothing was helpful were more likely to have more severe GWI (SPSS version 25). The independent variable was the three larger categories: (1) acknowledgement and validation; (2) specific recommendations for managing GWI or its symptoms (3) nothing a provider has said has been helpful. Each patient was only allowed one code – for patients whose responses had two codes, we chose the code that was the primary theme of the response. The dependent variables were greater disability (WHO-DAS 2.0), more severe physical symptoms (PHQ-15) and greater utilization of primary care. Statistical significance was set at *p* < .05.

## Results

The mean age of participants was 51.7 years (range 42–79 years), 23 (11.9%) were female; 8 (4.1%) identified as American Indian/Alaska Native, 4 (2.1%) as Asian, 45 (21.3%) as Black or African American, 131 (62.1%) as White, 14 (7.4%) as Hispanic or Latino/Latina; 7 (3.3%) as more than one race, and 1 (0.5%) as unknown; 90 (46.4%) were employed full-time, 7 (3.6%) were employed part-time. The average level of disability was 46.13 (18.9). The average level of physical symptom severity was 14.62 (4.88) which is considered high. Most patients had 2–3 visits (*n* = 82; 41.8%) or 4–9 visits per year (*n* = 66; 33.7%).

**Fig. 1:**
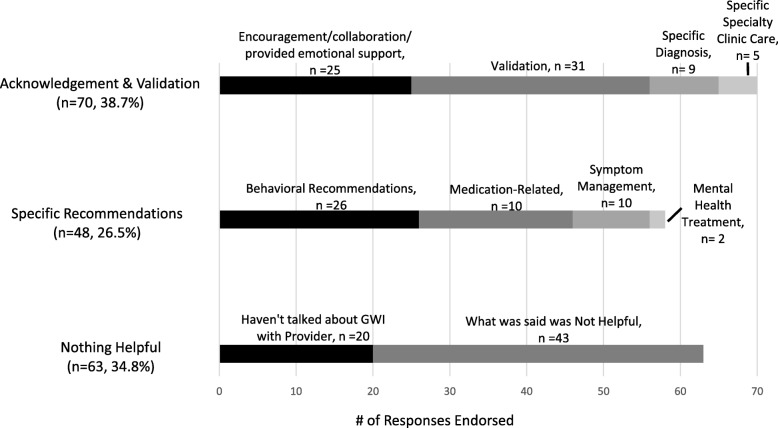
Frequency of coded responses

A total of 155 Veterans (92.3%) had responses that were categorized with a single code; 13 Veterans (7.7%) had responses that were coded with 2 codes. This resulted in 181 coded responses from 168 respondents. See Fig. 1 for the frequency of coded responses.

### Acknowledgement and validation

The most prevalent category was acknowledgment and validation (*n* = 70, 38.7% of 181 responses). There were four codes within acknowledging and validation. First, patients described providers giving encouragement/collaboration/emotional support (*n* = 25). Examples of this included “that it takes time to get back to normalcy, one step at a time and use action for situations”, “keep busy and stay positive”, “we have to try everything before we give up”, and “[you] can live with it. [you] can still have a good, meaningful life.” Second, patients described validation as useful (*n* = 31). Examples included “that my condition is real and not just a mental issue”, “they are checking and I am not alone. Others are suffering too.”, and “I believe you … [it] is not of [your] imagination.” Third, patients described being given a specific diagnosis as helpful (*n* = 9) such as “today…I was told I have GWI instead of Dr.’s telling me I was making it up.” Finally, five patients described the care they received at a specific specialty clinic as most helpful, for example: “what was shared with me in New Jersey”.

### Specific recommendations for managing GWI or its symptoms

Many respondents stated that providers gave them specific recommendations that were helpful (*n* = 48, 26.5% of 181 responses). There were four codes of specific helpful recommendations: behavioral recommendations, medication-related, symptom management and mental health treatment. Behavioral recommendations (*n* = 26) was the most common code. Examples include a wide-range of suggestions such as “get hobbies and other interests to take my mind off of it”, “start a protein diet to lose weight and lower blood sugar levels”, and “find sources to relax (breathing techniques, medication, exercise)”. In the medication-related code (*n* = 10), patients described receiving advice to take medications to help with specific symptoms. One patient stated a provider told him “that medical marijuana may be helpful in easing my nerve related and stomach related pain;” another patient described being told “not to use narcotics to treat possible GWI symptoms”. Patients also described receiving advice on symptom management or how to manage their GWI (*n* = 10) including learning that their “symptoms wax and wane”. The final category was mental health treatment (*n* = 2) an example included advise to “[stick] with counseling sessions”.

### Nothing a provider has said has been helpful

Many responses indicated that the provider had not said anything helpful about GWI (*n* = 63, 34.8% of 181 responses). There were two codes within this category. Many patients said that they haven’t talked about GWI with their provider (*n* = 20). Examples of this included: “have not talked to a provider in regards to GWI”, and “does not address my disabilities as GWI as a whole; they address each concern individually and I suspect don’t see the bigger picture”, “hasn’t been mentioned”. The second code was what the provider did say was not helpful (*n* = 43). Examples of this included: “nothing, I haven’t found one that believes in it”, and “haven’t had any say anything useful. I have only been told it is in my head and nothing is wrong with me by my VA doctors”.

### ANOVA analyses

Contrary to expectations, there were no differences in the severity of GWI among patients who responded that the most helpful communication was (1) acknowledgement and validation, (2) specific recommendations for managing GWI or its symptoms, or that (3) nothing a provider has said has been helpful. There were no statistically significant differences in level of disability (*F*(2,155) = 1.54, *p* = .22), severity of physical symptoms (*F*(2, 156) = .87, *p* = .42), or frequency of prior healthcare utilization (*F*(2, 159) = .14, *p* = .87).

## Discussion

The goal of this study was to identify patients’ perspectives of the most useful thing a medical provider has told them about their MUS. We examined this question among 210 Veterans with Gulf War Illness (GWI), a type of MUS that is marked by significant disability and for which there has been documented distrust of medical providers [21–24]. We found three primary themes: acknowledgement and validation, specific recommendations for managing GWI or its symptoms and that nothing a provider has said has been helpful.

The most prevalent category was acknowledgement and validation (*n* = 70) including validation that the symptoms were real and collaborative encouragement. This finding is important because qualitative studies have found that providers feel helpless and do not have the knowledge needed to treat MUS, but nonetheless feel pressured to provide a clear diagnosis and medical treatment [12, 33–35]. Our findings suggest that providers don’t need to have an answer to be helpful. Acknowledgement and respect may be the first step towards building an on-going collaborative relationship.

Communication of self-management strategies and specific treatment recommendations were also endorsed by a substantial proportion of the sample, this is consistent with prior studies that finds patients are looking for communication that is actionable [19]. Our results highlight the range of interventions patients find helpful including behavioral recommendations, medication-related, symptom management and mental health treatment. This supports a holistic approach to the care of patients with MUS who are interested in treatment options beyond medication. Of note, only two participants cited mental health treatment as the most useful recommendation, despite cognitive behavioral therapy being listed as a first line treatment recommendation for GWI and MUS more generally [36]. Providers may want to examine how they are delivering this recommendation in order for more patients to perceive it as helpful and supportive.

Overwhelmingly, the research on provider-patient communication around MUS suggests that both patients and providers report dissatisfaction. Consistent with previous work, we found that many of the patients (*n* = 63, 34.8%) indicated that nothing helpful was said by their provider about their GWI. Sometimes this was because what was said was perceived has unhelpful and counterproductive. Other times this was because the patient reported that GWI was not discussed at all. Indeed, research has found that patients may not bring up their MUS concerns for fear that the symptoms will be seen as a psychological problem. From the providers’ perspective, the opportunity to address MUS may not be obvious and they may miss patients’ cues to talk about MUS [37]. Our results are consistent with these findings and suggest that there are missed chances for providers to talk about patients’ symptoms.

Burton and colleagues have proposed that effective communication/treatment for MUS includes recognition (i.e., acknowledgement), explanation and action (i.e., specific treatment recommendations) [38]. Consistent with this, we found that patients report that acknowledgement and specific recommendations were helpful. We did not find patients reported an explanation as most helpful, with only nine patients describing a diagnosis of GWI (or another MUS) as most helpful and none describing a functional explanation of symptoms (e.g., overactive nerves) [39] as most helpful. This does not mean that an explanation isn’t an important component of effective patient-provider communication for MUS; providing an explanation may have laid the groundwork for positive reception of acknowledgement and specific recommendations. Alternatively, it may be that very few patients were given explanations, but those few were most satisfied. This should be examined in future studies.

We did not find that perceptions of helpful communication were related to patient-level differences in the severity of GWI, suggesting that dissatisfaction in communication is not due to some patients having more severe symptoms or greater disability. This finding is hopeful. It suggests that even patients with the most severe presentations are likely to find acknowledgement and specific treatment recommendations to be helpful.

A strength of this study is we examined perceptions of helpful communication among patients who historically have had complex relationship with providers. A limitation is we do not know what providers actually said. Therefore, we don’t know if acknowledgement and validation was viewed as more often helpful or was simply more frequently communicated as compared to specific recommendations or explanations. Further, while we didn’t limit the quantity of the responses, we only asked one question and patients had to write their response likely limiting the richness of responses – typically responses were one sentence or a single phrase. Finally, this is a sample of largely male Gulf War Veterans with GWI, a specific type of MUS. Additional research is needed to understand the generalizability of the findings.

## Conclusions

We found nearly two-thirds of patients reported one or more example of provider communication as helpful around their MUS. Although much has been said about difficulties in patient provider communication around MUS, we found most patients are able to identify at least some helpful aspects of their communication with providers. Importantly, acknowledgement and validation were commonly seen as helpful, suggesting that even if a provider doesn’t have a diagnosis or treatment recommendation, there are still useful ways to communicate.

While most participants cited helpful aspects of communication with their provider, a substantial portion noted never receiving helpful information. This suggests a real opportunity to recalibrate communication around MUS with patients, as well enhance training on how to communicate empathically and effectively with patients with MUS.

## Abbreviations

MUS: Medically unexplained illnesses/symptoms
PST: Problem-solving therapy
PPS: Persistent physical symptoms
GWI: Gulf war illness
